# A Mucin-Like Protein Is Essential for Oviposition in *Nilaparvata lugens*

**DOI:** 10.3389/fphys.2019.00551

**Published:** 2019-05-15

**Authors:** Yi-Han Lou, Yan Shen, Dan-Ting Li, Hai-jian Huang, Jia-Bao Lu, Chuan-Xi Zhang

**Affiliations:** State Key Laboratory of Rice Biology and Ministry of Agriculture Key Laboratory of Agricultural Entomology, Institute of Insect Science, Zhejiang University, Hangzhou, China

**Keywords:** brown planthopper, *Nilaparvata lugens*, mucin-like protein, eggshell, oviposition

## Abstract

Mucins play a variety of roles; for example, in vertebrates, mucins lubricate epithelial surfaces and protect tissue from physical and biological damage, however, knowledge of insect mucins is limited. Here, we identified an eggshell-related mucin-like protein, NlESMuc, in the brown planthopper (BPH), *Nilaparvata lugens*. *NlESMuc* was specifically expressed in the follicular cells from the egg chambers of the ovarioles. RNA interference (RNAi) was used to perform functional analysis of *NlESMuc*. Adult female BPH with *NlESMuc* knockdown had significantly reduced fecundity, including more difficult oviposition, lower egg production, and eggs that could not hatch. Scanning electron microscopy showed that, in *NlESMuc* knocked-down BPH, the ultrastructure of the eggshells of fully developed oocytes was loose, and the cross-section showed many small droplets of about 0.1-μm diameter. Based on the results, it is concluded that *NlESMuc* is an eggshell-related protein and essential for normal oviposition. Our findings help to provide new targets for pesticide design and RNAi-based BPH control and will also provide new insights into insect eggshells and insect mucins.

## Introduction

The brown planthopper (BPH), *Nilaparvata lugens*, is one of the most serious rice pests in Asia. It feeds exclusively on rice, damaging rice directly by sucking plant sap and by transmitting two viruses, rice ragged stunt virus and rice grassy stunt virus. The r-strategic BPH is a migratory insect, has a short lifespan, and shows high fecundity with no parental care. In appropriate conditions, BPHs have the potential to achieve high population numbers and cause leaves to initially turn orange-yellow before they become brown and dry, a phenomenon called hopperburn that kills the plant (Bao and Zhang, 2019).

Mucins are a group of high–molecular weight glycoproteins that are abundant in vertebrate respiratory and digestive tract, and are classified as membrane-associated or secreted mucins (Wagner et al., 2018). Mucins play a variety of roles, such as lubricating epithelial surfaces, protecting tissue against physical and biological damage (Syed et al., 2008), protecting proteins and cells from proteolysis (Jentoft, 1990), modulating cell attachment, and inhibiting immune cell function (Gimmi et al., 1996). Mucins are either membrane-associated or secreted. Although their molecular weights and protein sequences vary considerably, mucins are characterized by extended, tandem-repeated sequences rich in proline (Pro), serine (Ser), and threonine (Thr) and that are heavily substituted by *O*-linked oligosaccharides. The *O*-glycosylated sites cover 30–90% of the protein sequence, and when they are glycosylated, the molecule can be envisioned as a bottle brush, with an outstretched polypeptide backbone densely covered by carbohydrate moieties. A growing number of mucins and mucin-like proteins have been identified in insects, and they are mainly distributed in the salivary glands, midgut and Malpighian tubule (Kramerov et al., 1996; Wang and Granados, 1997; Sarauer et al., 2003; Syed et al., 2008; Huang et al., 2017). However, knowledge of mucin functions in non-mammalian animals is limited.

In BPH, abundant amounts of a mucin-like protein have been found in both the gelling and watery salivary proteomes (Huang et al., 2016, 2017). The start point of this work was identifying additional mucin-like proteins in *N. lugens*. We screened the genes annotated as mucin-like proteins in the *N. lugens* transcriptome database, of which one, designated *NlESMuc*, is exclusively expressed in the ovaries. There have been few studies of ovarian-specific insect mucin, which has aroused our interest. RNA interference (RNAi) revealed that *NlESMuc* was essential for oviposition. As BPH outbreaks can be partly explained by their high fecundity, the present study helps provide new targets for pesticide design and RNAi-based BPH control. It will also provide new insights into insect eggshells and mucins.

## Materials and Methods

### Insect

The BPH used in this study was collected from the Huajiachi Campus of Zhejiang University in Hangzhou, China. The BPHs were reared on rice variety Xiushui 134 which is susceptible to our BPH population in the artificial climate chamber. The parameters are set as follows: temperature 28 ± 0.5°C, humidity 45–55%, light/dark photoperiod 16/8 h. As brachypterous morphs usually have higher fecundity and earlier development of the ovaries than the macropterous morphs in many insects including *N. lugens* (Zera and Denno, 1997), brachypterous insects were used in this work.

### Sequence Analysis

Based on transcriptomic and genomic annotation (Xue et al., 2010, 2014), *NlESMuc* was cloned and sequenced. Signal peptide was predicted by SignalP server^1^ and conserved domains were predicted with HMMER^2^ with default parameters. The NetNGlyc 1.0 Server^3^ and NetOGlyc 4.0 Server^4^ were used to predict the *N*-glycosylation sites and *O*-glycosylation sites, respectively. ProtParam was used to compute the molecular weight, theoretical pI and amino acid composition^5^.

### Quantitative Real-Time PCR Analysis

Total RNA was isolated using RNAiso Plus (Takara, Dalian, China) according to manufacturer’s protocol. The quality and concentration of the RNA was analyzed by a NanoDrop 2000/2000c Spectrophotometer (Thermo Fisher Scientific, Bremen, Germany). First-strand cDNA was synthesized using ReverTra Ace qPCR RT Master Mix with a gDNA Remover Kit (ToYoBo, Osaka, Japan). Primers specific to *NlESMuc* were designed by the Primer Premier 6.0 program (PREMIER Biosoft, Palo Alto, CA, United States) (Supplementary Table S1). The *N. lugens* housekeeping genes 18S rRNA (GenBank accession number: JN662398.1) and ribosomal protein S11 (GenBank accession number: ACN79505.1) were used as the reference genes. The real-time PCR was conducted by a CFX96^TM^ Real-Time PCR Detection System (Bio-Rad, Hercules, CA, United States) under the following conditions: denaturation for 3 min at 95°C, followed by 40 cycles at 95°C for 10 s and 60°C for 30 s. The expression level of the target gene was normalized to the reference genes and calculated using the ΔΔCt method. Three biological replicates were performed, and each replicate was conducted three times (technical replicates).

### Tissue Dissection and Sample Collection

Dissection was conducted under an S8 APO stereomicroscope (Leica, Wetzlar, Germany). The insects were anesthetized on ice and placed on a Petri dish, and carefully dissected in phosphate-buffered saline (PBS; 0.9% NaCl, 0.02% KCl, 10 mM Na_2_HPO_4_, 2 mM KH_2_PO_4_, pH 7.4) using forceps. The separated tissues were washed three times with PBS and collected for RNA isolation or for morphological observation.

To investigate the development expression patterns of *NlESMuc*, total RNA was extracted from whole insects/eggs at various developmental stages, Specifically, eggs (*n* = 200) were collected every 24 h after laying, first and second instar nymphs (*n* = 50 for each) were collected at 0 h and 36 h after hatching or molting, third and fourth instar nymphs (*n* = 30 for each) were collected every 24 h after molting, and fifth instar nymphs (*n* = 30) and brachypterous adult male (*n* = 30) and female (*n* = 30) BPHs were collected every 12 h after molting/emergence. To investigate the tissue-specific expression pattern, The digestive tract (*n* = 50), Malpighian tubes (*n* = 50), salivary glands (*n* = 80), fat bodies (*n* = 50), ovipositors (*n* = 30), ovaries (*n* = 30) and integument (*n* = 20) of brachypterous females were dissected 48–120 h after emergence, while the testes (*n* = 30) were dissected from brachypterous male adults 48–120 h after emergence. Ovaries (*n* = 60) were further divided into terminal filament and germarium, small follicles (oocytes < 800 μm and surrounding follicular cells), basal oocyte (oocyte about 850 μm in length), follicular cells around the basal oocytes, and the remaining part (mainly oviduct). These samples were used as templates for quantitative reverse transcription–PCR (RT-qPCR) analysis.

### dsRNA-Induced RNA Interference

RNA interference (RNAi) was conducted according to Xue et al. (2015). Primers containing the *T*7 promoter were designed. The amplicons were cloned into pMD19-T vectors and the recombinant plasmids were used as templates for dsRNA synthesis with a MEGAscript T7 transcription kit (Ambion, Austin, TX, United States). Newly emerged female adult BPHs were injected with ∼250 ng dsNlESMuc (320 bp) or dsGFP (715 bp) at the junction between mesopedes and metapedes. About 50 female adults were used for injection with dsRNA targeting each gene in one treatment and three independent experiments were conducted. Seventy-two hours after dsRNA injection, total RNA was isolated from five individuals to check the transcript levels of *NlESMuc* by RT-qPCR.

To evaluate the fecundity and hatchability, injected female adult BPHs were transferred to glass tubes containing three rice seedlings 2 h post-injection, and each female was matched with two males of the same age. The insects were moved to a new tube every 3 days, and newly hatched BPHs were counted daily on day 6–10 after the parents had been removed, and then the remaining eggs that failed to hatch were counted under a microscope. Ten biological replicates were performed. To test the effect of humidity on hatching, one group of glass tubes was covered with plastic sheets to maintain high humidity, and another was covered with mesh fabric. The dsRNA-injected adult female BPHs (*n* = 20 for each group) were reared on rice seedlings with the same number of wild type male BPHs. To observe the morphology of the ovaries, the ds*NlESMuc* and ds*GFP* females were dissected on day 3–9 post-injection (*n* = 5 for each day). In addition, the duration for females in both groups to lay an egg was recorded. The ds*NlESMuc* and ds*GFP*-injected females were placed on rice seedlings in two glass tubes with wild type males, respectively. The oviposition behavior was observed under naked eyes and the time from ovipositor penetration to withdrawal was measured by the same person using a stopwatch.

### Electron Microscope Observation

Samples were prepared by dissecting adult females from ds*NlESMuc*-injected and ds*GFP*-injected groups 4 days after emergence. The oocytes were dissected in PBS and fixed in 4% [volume per volume ratio (v/v)] glutaraldehyde in PBS at 48°C overnight. For the oocytes cross sectioned with forceps, additional fixation in 4%(v/v) glutaraldehyde in PBS at 48°C overnight was needed. The samples were washed three times in PBS and post-fixed with 1% (v/v) osmium tetroxide for 1.5 h at room temperature. The fixed oocytes were dehydrated via immersion in a graded series of ethanol (50, 70, 80, 90, 95%, v/v) for 15 min each, then transferred into 100% ethanol for 30 min and repeated once, then immersed in ethanol/isoamyl acetate (v/v = 1/1) for 30 min, and placed in isoamyl acetate overnight. The samples were dried in a desiccator under a vacuum and attached to a stub for gold sputtering. The samples were observed under TM-1000 Scanning Electron Microscope (THitachi, Tokyo, Japan).

### Statistical Analysis

Data are presented as mean ± SEM or mean ± SD as noted in the figure legends. Statistical analysis was performed using GRAPHPAD PRISM 7.0 (GraphPad Software, La Jolla, CA, United States). Differences between two groups were compared using a two-tailed unpaired *t*-test at the significance levels of ^∗^*P* < 0.05 and ^∗∗^*P* ≤ 0.001. One-way analysis of variance (ANOVA) was applied for comparing the differences among three or more samples.

## Results

### Sequence Analysis

**FIGURE 1:**
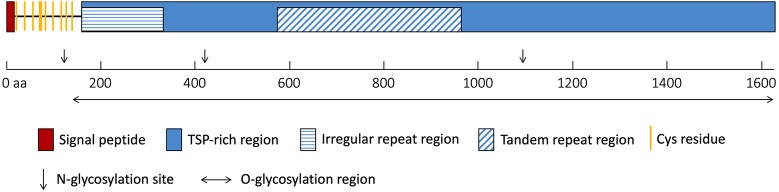
Schematic of *NlESMuc* protein sequence. The polypeptide is drawn to scale and key features are indicated. TSP-rich region, threonine/serine/proline-rich region. aa, amino acids.

The *NlESMuc* complementary DNA (cDNA) sequence was obtained from the *N. lugens* transcriptome database, cloned, and was verified by sequencing. The open reading frame of *NlESMuc* (GenBank accession number: MK693138) contained 4881 bp and encoded a 1626–amino acid protein. The calculated molecular weight and theoretical isoelectric point (pI) was 158.1 kDa and 5.53, respectively. The first 16 amino acids were predicted to be the signal peptide. The sequence contained a Thr/Ser/Pro (TSP)-rich region, which contained an irregular repeat region and a tandem repeat region (Figure 1). The tandem repeat region consisted of 21 base pair repeats encoding a 7–amino acid repeating peptide (SPSSTTA). There were 10 cysteine residues, which could form five intramolecular disulphide bridges, between the signal peptide and the TSP-rich region. Three *N*-glycosylation sites were found irregularly, while 1012 potential *O*-glycosylation sites were predicted throughout the sequence except in the first 135 amino acids. Given its high TSP content, tandem repeat sequences, and heavy glycosylation, *NlESMuc* was considered a mucin-like protein in BPH.

### Expression Pattern

**FIGURE 2:**
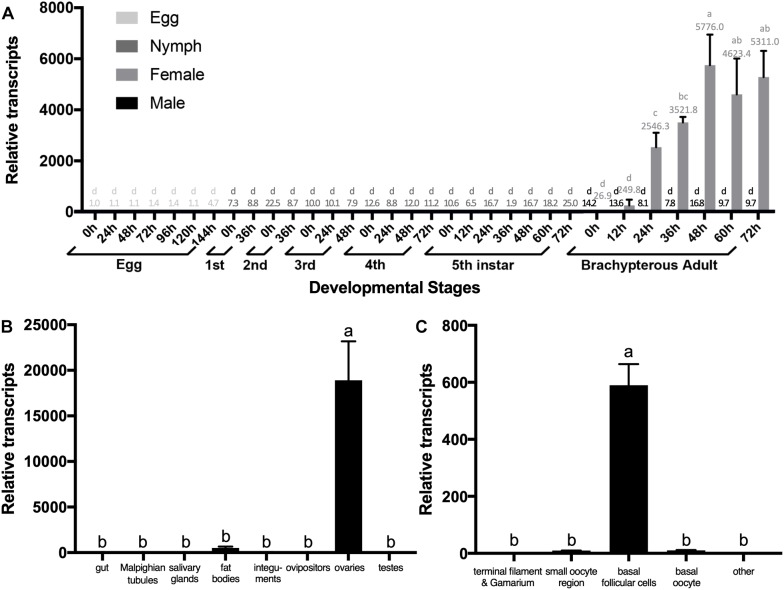
The expression pattern of *NlESMuc*. **(A)** NlESMuc expression according to BPH developmental stage. Samples were collected from eggs, nymphs, and brachypterous male and female adults. The *NlESMuc* mRNA level in eggs at 0 h was designated 1 and relative expression levels for each sample were marked in different colors according to the color code. **(B)** NlESMuc expression in BPH tissues. The digestive tract (*n* = 50), Malpighian tubes (*n* = 50), salivary glands (*n* = 80), fat bodies (*n* = 50), ovipositors (*n* = 30) and integument (*n* = 20) of brachypterous females were dissected 48–120 h after emergence, while the inner reproductive organs (*n* = 30), testes (male), and ovaries (female) of brachypterous adults were dissected 48–120 h after emergence. The *NlESMuc* mRNA level in the integument was designated 1. **(C)** *NlESMuc* expression in BPH ovary. The terminal filament–germarium region, small oocyte region (containing small developing oocytes < 800 μm and active follicular cells), basal oocytes (oocytes ∼850 μm in length), basal follicular cells (follicular cells around the basal oocytes), and other parts (oviduct, bursa copulatrix, spermatheca, pouched gland) were dissected from brachypterous adult females 48–120 h after emergence. The *NlESMuc* mRNA level in the other parts was designated 1. Nl18S and RPS11 were used as internal control genes. Data represent the relative *NlESMuc* mRNA levels. Data are the mean ± SEM from three independent experiments. Columns followed by a common letter are not significantly different by the HSD-test at the 5% level of significance in each histogram.

We quantified relative transcripts of *NlESMuc* in different BPH developmental stages, namely egg/embryo, nymph, and adult (Figure 2A). *NlESMuc* was expressed specifically in adult female BPH and its expression began 24 h post-emergence. The *NlESMuc* expression levels in the other developmental stages were comparable to that in adult male BPH.

To indicate the potential role of *NlESMuc*, we examined its expression levels in different tissues (Figure 2B). As *NlESMuc* mRNA expression was female-specific, we dissected six parts, i.e., the digestive tract, internal reproductive organs (ovaries), ovipositors, integument, salivary glands, and fat bodies, of adult female BPHs. The testes of male adult BPHs were also dissected. When compared in the seven tissues mentioned above, *NlESMuc* expression was highest in the ovaries.

To locate *NlESMuc* in the ovaries, the basic unit of ovaries, i.e., the ovariole, was divided into four parts: terminal filament and germarium region, small oocyte region, basal follicular epithelium cells, and basal oocytes. The other female BPH internal reproductive organs were the oviduct, bursa copulatrix, spermatheca, and pouched gland. In these five parts, *NlESMuc* mRNA level was highest in the basal follicular epithelium cells (Figure 2C), which was similar to mammalian mucins that produced by epithelial tissues.

### RNAi

The *NlESMuc* expression pattern indicated that it might play an important role in reproduction. To investigate the function of *NlESMuc*, we conducted RNAi experiments through microinjection of specific double-stranded RNA (dsRNA) targeting *NlESMuc* (ds*NlESMuc*) into newly emerged brachypterous adult female BPHs. The control group was injected with dsRNA corresponding to the gene for green fluorescent protein (ds*GFP*).

**FIGURE 3:**
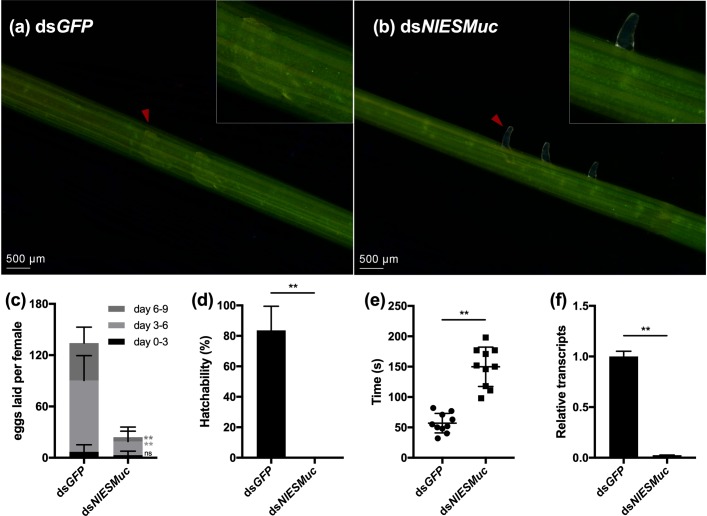
The effect of RNAi on oviposition in dsGFP (control) and ds*NlESMuc* groups. **(a,b)** The eggs laid in rice seedlings; the upper right corner shows an enlarged image. Arrowheads indicate the eggs laid in both groups. The ds*NlESMuc* eggs were partially inside the rice tissue, leaving the anterior half and micropyle area exposed. **(c)** The average number of eggs laid per female in both groups. The dsNlESMuc females had significantly reduced fecundity. **(d)** The hatchability in both groups. The hatchability in ds*NlESMuc* group was significantly affected when compared to that in dsGFP group. **(e)** The duration from ovipositor penetration to withdrawal. The process was longer after *NlESMuc* knockdown. **(f)** RNAi efficiency. Not significant (ns) *P* ≥ 0.05, ^∗∗^*P* ≤ 0.001.

Interestingly, the inhibition of *NlESMuc* resulted in abnormal oviposition. In general, the ovipositor penetrated the plant epidermis and inserted eggs into the plant tissue, and then the ovipositor was retracted from the plant for the next oviposition. However, in the ds*NlESMuc* group, eggs were laid partially inside the rice tissue, leaving the anterior half and micropyle area exposed (Figure 3a,b), and fewer eggs were laid compared to the control group (Figure 3c). In the first 3 days after emergence, very few eggs were laid in both groups and showed no significant difference (*p* = 0.11) since females just started to oviposit. On days 3 to 6 and days 6 to 9, the number of eggs laid in the ds*NlESMuc* group was significantly less than the control group (*P* < 0.001). In addition, none of the eggs laid by the ds*NlESMuc* females hatched in 45–55% humidity during the observation period, while 83.62% of control eggs laid hatched (Figure 3d).

We recorded the duration of ovipositor penetration to withdrawal. The process spanned an average 57.0 s for ds*GFP* adult female BPHs; in the ds*NlESMuc* group, the mean duration was 149.8 s (Figure 3e), which was significantly longer than that in the ds*GFP* group (*P* < 0.001). We speculate the extended oviposition time is due to the difficulty of the egg moving through the oviduct and/or ovipositor. Considering *NlESMuc* was highly expressed in the follicular cells other than oviducts, its function may be related to the eggshell covering the oocyte surface.

Efficient knockdown of *NlESMuc* expression after injection of the dsRNAs was confirmed by RT-qPCR (Figure 3f).

**FIGURE 4:**
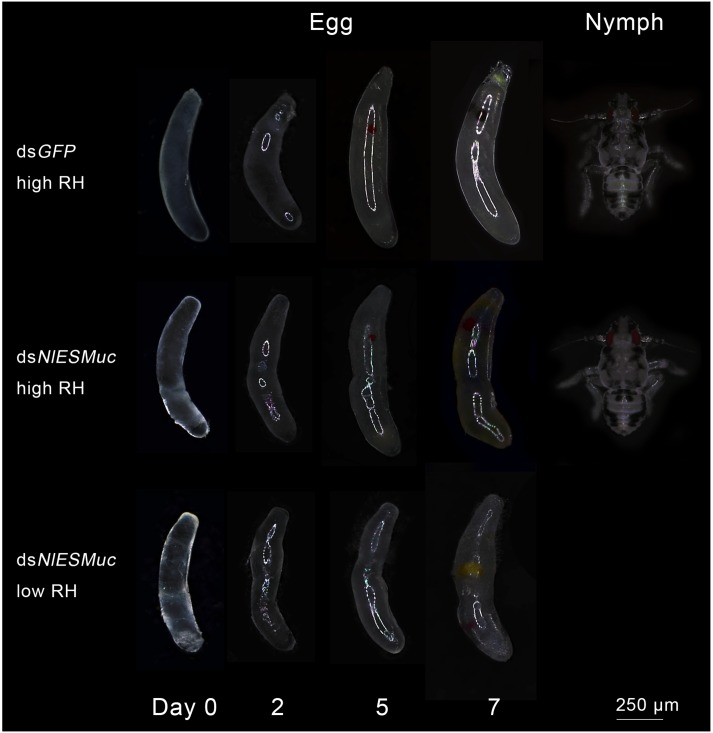
Egg development in dsGFP (control) and dsNlESMuc groups. Different humidity levels were set, and each treatment contained 20 dsRNA-injected females. Normal BPH eggs were cylindrical and slightly curved, while the dsNlESMuc eggs dissected from the rice seedlings were irregularly shaped. dsNlESMuc eggs kept in 95–100% relative humidity could hatch successfully, but almost all dsNlESMuc eggs in 45–55% relative humidity failed to hatch. RH, relative humidity.

To determine whether the hatching failure of ds*NlESMuc* eggs resulted from water loss by the eggs or that they could not be properly fertilized, glass tubes containing rice seedlings with oviposited eggs were covered with plastic sheets to maintain 95–100% relative humidity. Here, the ds*NlESMuc* eggs hatched successfully (Figure 4), indicating that *NlESMuc* interference did not affect fertilization. The low hatchability may have been due to dehydration following the exposure of the eggs to the external environment or changes in eggshell permeability.

**FIGURE 5:**
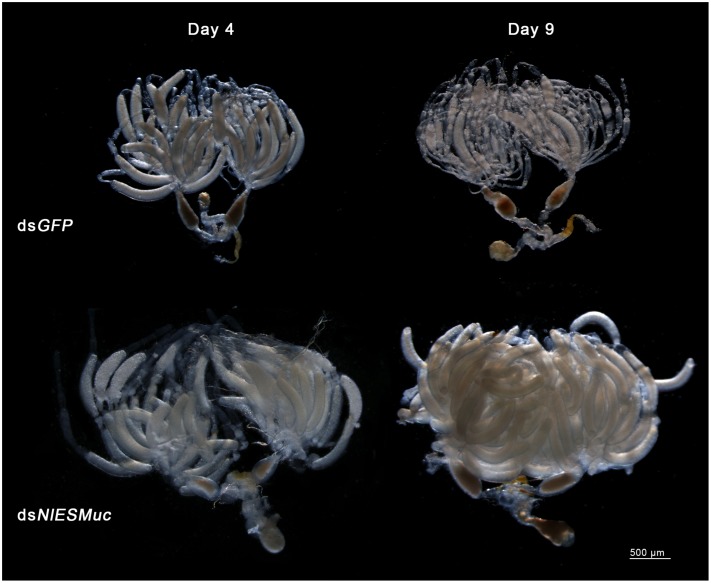
The effect of RNAi on the ovaries in the dsGFP (control) and dsNlESMuc groups. There was no significant difference in oocyte morphology between the two groups. The ovaries in both groups contained considerable developed oocytes on day 4 after emergence. On day 9, ovaries in the dissected dsNlESMuc females (*n* = 5) were full of developed oocytes, while most of the developed oocytes in the control group were oviposited.

Brown planthopper eggs are cylindrical and slightly curved, but the ds*NlESMuc* eggs dissected from the rice seedlings were irregularly shaped (Figure 4). Dissection of the female BPH ovaries showed that oocytes in the ds*NlESMuc* group had normal morphology (Figure 5); therefore, the rice tissue caused the egg denting. In addition, on day 9 after emergence, ovaries dissected from ds*NlESMuc* females remained full of developed oocytes; in the control group, most of the developed oocytes were oviposited (Figure 5).

### Electron Microscope Observation

**FIGURE 6:**
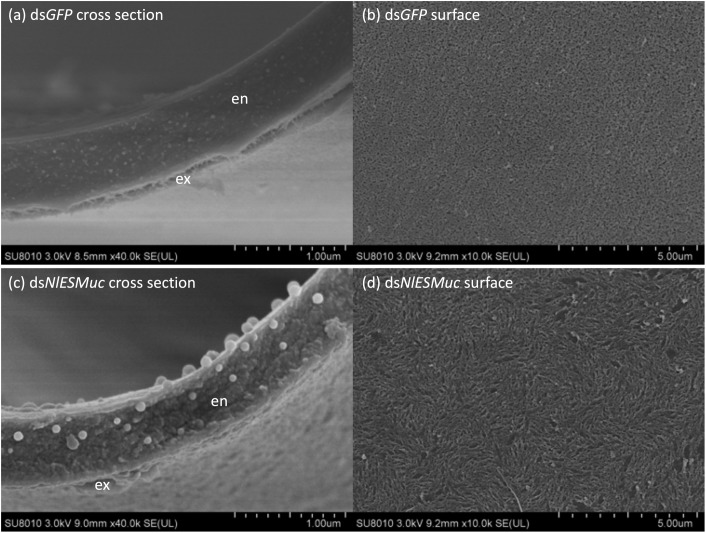
Scanning electron microscope observation of developed oocytes from dsGFP (control) and dsNlESMuc adult female BPH. **(a,c)** Cross-section of the developed oocytes (*n* = 3). All observed dsNlESMuc eggshells had a looser structure, and there were many small droplets of ∼0.1-μm diameter. En, endochorion; ex, exochorion. **(b,d)** The surface of developed oocytes (*n* = 5). dsNlESMuc oocytes had rougher surfaces.

To determine whether *NlESMuc* plays an important role in the eggshell formation, we used electron microscopy to observe the ultrastructure of eggshells from the ds*NlESMuc* and ds*GFP* groups. The eggshells of ds*NlESMuc* oocytes had a looser structure, and there were many small droplets about 0.1 μm in diameter (Figure 6a,c). In addition, the oocytes had rougher surfaces, and some even had irregular burrs on the surface (Figure 6b,d). Taken together, these results suggest that *NlESMuc* is associated with eggshell formation.

## Discussion

We investigated the potential role of a mucin-like protein, *NlESMuc*, in BPH. The gene encoding *NlESMuc* is only expressed in adult female BPH and showed an ovary-specific expression pattern. Silencing *NlESMuc* caused abnormal oviposition behavior, that is, the anterior part of the eggs remained outside the rice tissue, and there was decreased egg production and prolonged egg-release time. During oviposition, the ovipositor penetrated into plant epidermis, followed by forward-backward motions. The forward-backward motion extends the cut downward to form a slit into which an egg could be inserted, and the egg is released after almost-full penetration of the ovipositor (Hattori and Sogawa, 2002). According to this, the phenomenon after RNAi may not be related to the hardness of the egg. A possible explanation for this might be that the eggs in ds*NlESMuc* females have difficulties passing through the ovipositor and being separated from the ovipositor with the help of rice tissues, which may be caused by rough and/or inelastic eggshells. Moreover, the cross-section of developed oocytes in *NlESMuc*-knockdown females showed a roughened eggshell structure under the scanning electron microscope, and extra, small droplets were observed. The result further supports the role of *NlESMuc* in eggshell formation.

*NlESMuc* has a similar expression pattern to the known eggshell protein *NlChP* (Lou et al., 2018), however, we found some differences. *NlChP* expression started about 48 h after emergence in female adult BPH, while *NlESMuc* expression started about 24 h after emergence, indicating earlier transcription of *NlESMuc*. *NlChP* expression was highest in the small oocyte region, which included developing oocytes and surrounding follicular cells, while *NlESMuc* expression was highest in the basal ooecium wall, which mainly consists of follicular cells around the basal oocyte. That is, *NlESMuc* is expressed even in the late stage of eggshell formation. The knockdown of *NlChP* did not affect the expression of *NlESMuc*, and vice versa (Supplementary Figure S1). However, after RNAi of forkhead box transcription factor L2, which regulates eggshell formation in *Nilaparvata lugens* (Ye et al., 2017), the mRNA level of *NlESMuc* decreased by 32.24% (*P* = 0.04) at 72 h after emergence (Supplementary Figure S1). These results indicate that *NlESMuc* might function in both the early and late stages of eggshell formation.

Mucins are characterized by high Ser/Thr/Pro content, tandem repeated sequences and heavy glycosylation. *NlESMuc* shares these features with mucins and was identified as a mucin-like protein. Mucins usually contain other domains, e.g., mammalian mucins have D domains and cystine-knot domains (Brown and Hollingsworth, 2013) and *Drosophila* mucins have peritrophin A (PerA) chitin-binding domains (Syed et al., 2008). However, we only found three regions sharing low sequence similarities to the PerA chitin-binding domain in the NlESMuc N-terminal. Another characterized mucin-like protein in BPH, NlMul, which is a saliva component, also has no other domains (Huang et al., 2017).

*O*-glycosylation is an evolutionarily conserved protein modification that was found in mammals, echinoderms, worms, insects, protozoa, and certain types of fungi (Tran and Ten Hagen, 2013). This modification is characterized by the attachment of a variety number of O-glycans via the linkage sugar N-acetylgalactosamine. O-glycans are often sialylated or sulfated and negatively charged (Thornton et al., 2008). The extensive *O*-glycosylation in mucins makes the protein form an extended rod-like structure (Shogren et al., 1989). And the dense oligosaccharide packing protects the glycosylated regions from proteolysis (Jentoft, 1990). These properties may help NlESMuc molecules, which containing 1012 potential *O*-glycosylation sites, form a network through physical bonds like hydrophobic interactions and the structure can be stabilized by electrostatic repulsion between the negatively charged polysaccharide side chains. Moreover, NlESMuc contains the cysteine-rich motifs at the N-terminal regions and has the potential to polymerize via end-to-end disulfide bonds to form even larger macromonomer chains that are arranged in a linear manner. Another type of glycoprotein, zona pellucida (ZP) glycoproteins, is expressed in the ovaries and surround mature ova in vertebrates. ZP glycoproteins are held together in fibrils by non-covalent interactions and constitute a matrix (Wassarman, 2008). The oligosaccharides of these glycoproteins are considered to play key roles in spermatozoa–egg recognition (Wassarman, 1995). However, eggs derived from ds*NlESMuc* female BPHs can be properly fertilized in our study. It was surmised that NlESMuc would involve in the formation of the eggshell framework, as cross-sectioned oocytes from the ds*NlESMuc* BPH showed small droplets that may be components of the eggshell, suggesting that they could not be assembled correctly into the eggshell. On the other hand, NlESMuc might play a role in lubricating oocyte surfaces. The detailed functions of NlESMuc require further work.

In summary, we report an ovary specific mucin-like protein, designated as NlESMuc, in the BPHs. The functional analysis by RNAi revealed that NlESMuc played an important role in oviposition and was identified as an eggshell-related protein. This work contributes to existing knowledge of invertebrate mucins and provides a potential target for RNAi-based pest control of BPH.

## Data Availability

The datasets for this manuscript are not publicly available because the gene sequence has been submitted to GenBank and the accession number is MK693138. It will be released to the public database as soon as it is processed. Requests to access the datasets should be directed to chxzhang@zju.edu.cn.

## Author Contributions

Y-HL and C-XZ conceived the experiments and wrote the manuscript. Y-HL performed the main experiments. YS, D-TL, H-JH, and J-BL helped to perform the experiments. All authors contributed to critical analysis and approved the final manuscript for publication.

## Conflict of Interest Statement

The authors declare that the research was conducted in the absence of any commercial or financial relationships that could be construed as a potential conflict of interest.

## Footnotes

1 http://www.cbs.dtu.dk/services/SignalP-4.1/

2 https://www.ebi.ac.uk/Tools/hmmer/search/hmmscan

3 http://www.cbs.dtu.dk/services/NetNGlyc/

4 http://www.cbs.dtu.dk/services/NetOGlyc/

5 https://web.expasy.org/protparam/

## Supplementary Material

FIGURE S1 Relationship between *NlESMuc* and NlChP/ FoxL2. **(a)** The expression level of a known eggshell protein gene NlChP in dsNlESMuc-injected female BPHs at 72 h post-emergence. There was no significant difference. **(b)** The expression level of *NlESMuc* in dsNlChP-injected female BPHs at 72 h post-emergence. There was no significant difference. **(c)** The expression level of *NlESMuc* after the RNAi of NlFoxL2, a transcription factor which regulates eggshell formation in BPHs, at 72 h post-emergence. The mRNA level of *NlESMuc* decreased by 32.24% (*p* = 0.04).

## References

[B1] Bao Y.-Y. Zhang C.-X. (2019). Recent advances in molecular biology research of a rice pest, the brown planthopper. *J. Integr. Agr.* 18 716–728. 10.1016/S2095-3119(17)61888-4

[B2] Brown R. B. Hollingsworth M. A. (2013). *Mucin Family of Glycoproteins*, 2nd Edn. Amsterdam: Elsevier Inc., 10.1016/B978-0-12-378630-2.00670-8

[B3] Gimmi C. D. Morrison B. W. Mainprice B. A. Gribben J. G. Boussiotis V. A. Freeman G. J. (1996). Breast cancer–associated antigen, DF3/MUC1, induces apoptosis of activated human T cells. *Nat. Med.* 2 1367–1370. 10.1038/nm1296-1367 8946837

[B4] Hattori M. Sogawa K. (2002). Oviposition behavior of the rice brown planthopper, *Nilaparvata lugens* (Stål), and its electronic monitoring. *J. Insect Behav.* 15 283–293. 10.1023/A:1015445202906

[B5] Huang H.-J. Liu C.-W. Huang X.-H. Zhou X. Zhuo J.-C. Zhang C.-X. (2016). Screening and functional analyses of *Nilaparvata lugens* salivary proteome. *J. Proteome Res.* 15 1883–1896. 10.1021/acs.jproteome.6b00086 27142481

[B6] Huang H.-J. Liu C.-W. Xu H.-J. Bao Y.-Y. Zhang C.-X. (2017). Mucin-like protein, a saliva component involved in brown planthopper virulence and host adaptation. *J. Insect Physiol.* 98 223–230. 10.1016/j.jinsphys.2017.01.012 28115117

[B7] Jentoft N. (1990). Why are proteins O-glycosylated? *Trends Biochem. Sci.* 15 291–294. 10.1016/0968-0004(90)90014-3 2204153

[B8] Kramerov A. A. Arbatsky N. P. Rozovsky Y. M. Mikhaleva E. A. Polesskaya O. O. Gvozdev V. A. (1996). Mucin-type glycoprotein from *Drosophila melanogaster* embryonic cells: characterization of carbohydrate component. *FEBS Lett.* 378 213–218. 10.1016/0014-5793(95)01444-6 8557103

[B9] Lou Y. H. Pan P. L. Ye Y. X. Cheng C. Xu H. J. Zhang C. X. (2018). Identification and functional analysis of a novel chorion protein essential for egg maturation in the brown planthopper. *Insect Mol. Biol.* 27 393–403. 10.1111/imb.12380 29465791

[B10] Sarauer B. L. Gillott C. Hegedus D. (2003). Characterization of an intestinal mucin from the peritrophic matrix of the diamondback moth, *Plutella xylostella*. *Insect Mol. Biol.* 12 333–343. 10.1046/j.1365-2583.2003.00420.x 12864913

[B11] Shogren R. Gerken T. A. Jentoft N. (1989). Role of glycosylation on the conformation and chain dimensions of O-linked glycoproteins: light-scattering studies of ovine submaxillary mucin. *Biochemistry* 28 5525–5536. 10.1021/bi00439a029 2775721

[B12] Syed Z. A. Härd T. Uv A. van Dijk-Härd I. F. (2008). A potential role for *Drosophila* mucins in development and physiology. *PLoS One* 3:e3041. 10.1371/journal.pone.0003041 18725942 PMC2515642

[B13] Thornton D. J. Rousseau K. McGuckin M. A. (2008). Structure and function of the polymeric mucins in airways mucus. *Annu. Rev. Physiol.* 70 459–486. 10.1146/annurev.physiol.70.113006.100702 17850213

[B14] Tran D. T. Ten Hagen K. G. (2013). Mucin-type O-Glycosylation during development. *J. Biol. Chem.* 288 6921–6929. 10.1074/jbc.R112.418558 23329828 PMC3591602

[B15] Wagner C. E. Wheeler K. M. Ribbeck K. (2018). Mucins and their role in shaping the functions of mucus barriers. *Annu. Rev. Cell Dev. Biol.* 34 189–215. 10.1146/annurev-cellbio-100617-062818 30296390 PMC11906035

[B16] Wang P. Granados R. R. (1997). An intestinal mucin is the target substrate for a baculovirus enhancin. *Proc. Natl. Acad. Sci. U. S. A.* 94 6977–6982. 10.1073/pnas.94.13.6977 9192677 PMC21270

[B17] Wassarman P. M. (1995). Towards molecular mechanisms for gamete adhesion and fusion during mammalian fertilization. *Curr. Opin. Cell Biol.* 7 658–664. 10.1016/0955-0674(95)80107-3 8573340

[B18] Wassarman P. M. (2008). Zona pellucida glycoproteins. *J. Biol. Chem.* 283 24285–24289. 10.1074/jbc.R800027200 18539589 PMC2528931

[B19] Xue J. Bao Y.-Y. Li B.-L. Cheng Y.-B. Peng Z.-Y. Liu H. (2010). Transcriptome analysis of the brown planthopper *Nilaparvata lugens*. *PLoS One* 5:e14233. 10.1371/journal.pone.0014233 21151909 PMC2997790

[B20] Xue J. Ye Y.-X. Jiang Y.-Q. Zhuo J.-C. Huang H.-J. Cheng R.-L. (2015). Efficient RNAi of rice planthoppers using microinjection. *Protoc. Exch.* 10.1038/protex.2015.005

[B21] Xue J. Zhou X. Zhang C.-X. Yu L.-L. Fan H.-W. Wang Z. (2014). Genomes of the rice pest brown planthopper and its endosymbionts reveal complex complementary contributions for host adaptation. *Genome Biol.* 15 521. 10.1186/s13059-014-0521-0 25609551 PMC4269174

[B22] Ye Y.-X. Pan P.-L. Xu J.-Y. Shen Z.-F. Kang D. Lu J.-B. (2017). Forkhead box transcription factor L2 activates Fcp3C to regulate insect chorion formation. *Open Biol.* 7:170061. 10.1098/rsob.170061 28615473 PMC5493777

[B23] Zera A. J. Denno R. F. (1997). Physiology and Ecology of Dispersal Polymorphism in Insects. *Annu. Rev. Entomol.* 42 207–230. 10.1146/annurev.ento.42.1.207 15012313

