# Supplementary material for: A Mucin-Like Protein Is Essential for Oviposition in Nilaparvata lugens
Source: Front Physiol. 2019 May 15;10:551. doi: 10.3389/fphys.2019.00551 (PMC6530529; doi:10.3389/fphys.2019.00551)
Supplement: FIGURE S1 — Relationship between NlESMuc and NlChP/ FoxL2. (a) The expression level of a known eggshell protein gene NlChP in dsNlESMuc-injected female BPHs at 72 h post-emergence. There was no significant difference. (b) The expression level of NlESMuc in dsNlChP-injected female BPHs at 72 h post-emergence. There was no significant difference. (c) The expression level of NlESMuc after the RNAi of NlFoxL2, a transcription factor which regulates eggshell formation in BPHs, at 72 h post-emergence. The mRNA level of NlESMuc decreased by 32.24% (p = 0.04). [file Table_1.pdf]

Table S1. Primers used in this work.

Primer usage	Primer name	Primer sequence (5'- 3')	Concentration
double stranded RNA synthesis	T7NlESMuc-F	TAATACGACTCACTATAGGGCAAACAGTAC AACTGCACCTCC	10mM
	T7NlESMuc-R	TAATACGACTCACTATAGGGGCTTGGCGAT ACTGTGCCAGG	10mM
	T7GFP-F	TAATACGACTCACTATAGGGAGAATGAGTA AAGGAGAAGAACTTTTC	10mM
	T7GFP-R	TAATACGACTCACTATAGGGAGATTTGTAT AGTTCATCCATGCCATGT	10mM
quantitative real-time PCR	qNlESMuc-F	AGTGGCATTCGTTCTTAAAGG	10mM
	qNlESMuc-R	TGTGGTTGTGGCTTGTGG	10mM
	RPS11-F	CCGATCGTGTGGCGTTGAAGGG	10mM
	RPS11-R	ATGGCCGACATTCTTCCAGGTCC	10mM
	18S-F	GTAACCCGCTGAACCTCCT	10mM
	18S-R	TCCGAAGACCTCACTAAATC	10mM

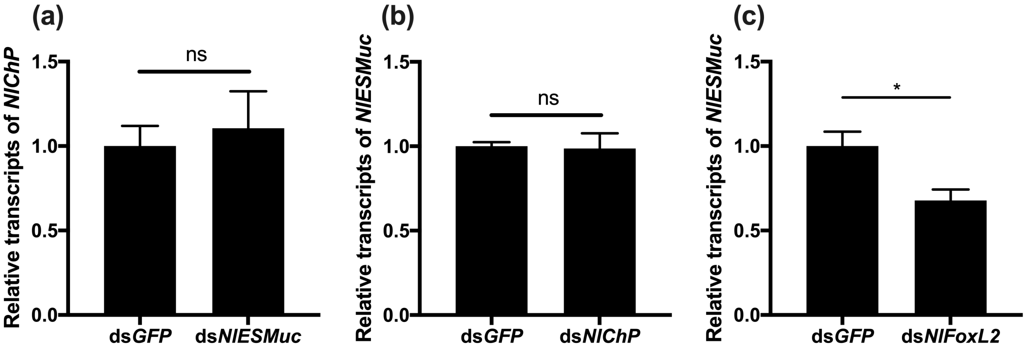

Figure S1. (a) The expression level of a known eggshell protein gene *NlChP* in ds*NlESMuc*-injected female BPHs at 72 h post emergence. There was no significant difference. (b) The expression level of *NlESMuc* in ds*NlChP*-injected female BPHs at 72 h post emergence. There was no significant difference. (c) The expression level of *NlESMuc* after the RNAi of *NlFoxL2*, which regulates eggshell formation in BPHs, at 72 h post emergence. The mRNA level of *NlESMuc* decreased by 32.24% ($p = 0.04$).
